# Comparing subsampling strategies for metagenomic analysis in microbial studies using amplicon sequence variants versus operational taxonomic units

**DOI:** 10.1371/journal.pone.0315720

**Published:** 2024-12-30

**Authors:** Daniel Segura, Divya Sharma, Osvaldo Espin-Garcia

**Affiliations:** 1 Department of Epidemiology and Biostatistics, University of Western Ontario, London, Ontario, Canada; 2 Department of Mathematics and Statistics, York University, Toronto, Ontario, Canada; 3 Dalla Lana School of Public Health, University of Toronto, Toronto, Ontario, Canada; 4 Department of Biostatistics, University Health Network, Toronto, Ontario, Canada; Stazione Zoologica Anton Dohrn, ITALY

## Abstract

The microbiome is increasingly regarded as a key component of human health, and analysis of microbiome data can aid in the development of precision medicine. Due to the high cost of shotgun metagenomic sequencing (SM-seq), microbiome analyses can be done cost-effectively in two phases: Phase 1-sequencing of 16S ribosomal RNA, and Phase 2-SM-seq of an informative subsample. Existing research suggests strategies to select the subsample based on biological diversity and dissimilarity metrics calculated using operational taxonomic units (OTUs). However, the microbiome field has progressed towards amplicon sequencing variants (ASVs), as they provide more precise microbe identification and sample diversity information. The aim of this work is to compare the subsampling strategies for two-phase metagenomic studies when using ASVs instead of OTUs, and to propose data driven strategies for subsample selection through dimension reduction techniques. We used 199 samples of infant-gut microbiome data from the DIABIMMUNE project to generate ASVs and OTUs, then generated subsamples based on five existing biologically driven subsampling methods and two data driven methods. Linear discriminant analysis Effect Size (LEfSe) was used to assess differential representation of taxa between the subsamples and the overall sample. The use of ASVs showed a 50-93% agreement in the subsample selection with the use of OTUs for the subsampling methods evaluated, and showed a similar bacterial representation across all methods. Although sampling using ASVs and OTUs typically lead to similar results for each subsample, ASVs had more clades that differed in expression levels between allergic and non-allergic individuals across all sample sizes compared to OTUs, and led to more biomarkers discovered at Phase 2-SM-seq level.

## Introduction

The human microbiome or ‘metagenome’ is characterized by the genetic material of the trillion microbial cells present in each individual [1–3]. From the moment of childbirth, the microbiome plays a key component in a human’s health [3–5]. Factors in an individual’s life, such as age, nutrition, and lifestyle changes greatly impact the composition of the human microbiome, as it continuously evolves in response to these changes. However, imbalance of the human microbiota (dysbiosis) has been linked to various health conditions such as cancer, cardiovascular disease, depression, respiratory illness, rhinitis pigmentosa and antibiotic-resistant bacterial infections [5–8].

The ability to analyse microbiome data quickly and reliably can guide decisions in the development of personalized precision medicine [9–11]. A person’s microbiome profile is a crucial part of the pharmacogenomic profile due to its contribution to the metabolism of certain drugs. A person’s microbiome profile could also be used to assess the immune system prior to immunotherapy and to guide patient stratification in clinical trials [12].

The most common approaches to characterise the microbiome typically target amplicons from taxonomic markers such as the 16S ribosomal RNA (16S rRNA) genes, a conserved region of DNA in bacteria. The 16S rRNA genes contain nine hypervariable regions which differ greatly among different bacterial species, allowing for their use in the identification of bacterial species via sequencing [13]. 16S rRNA sequencing data allows us to identify the microbial composition of a particular sample, but more is needed to extract the functional capabilities of a particular sample [9, 11].

One method used to obtain more comprehensive metagenomic information is shotgun metagenomic sequencing (SM-seq). Though there are methods that can obtain this information from the 16s rRNA, such as PICRUSt2, these methods are comparable to SM-seq [14]. However, it is typically unfeasible to perform SM-seq on numerous samples due to the high sequencing cost. The specific costs of SM-seq constantly changes but based on the commercial prices of various labs and institutions the costs of 16S rRNA sequencing versus shotgun metagenomic sequencing are $30 versus $300 per sample, respectively [15, 16]. The expenses of SM-seq can be mitigated by performing microbial research in two phases: First, the 16S rRNA of the microbiome of all participants is sequenced, these data is then used (possibly in combination with a phenotype of interest) to generate an informative subsample [5]. Second, the microbiome of the individuals in the informative subsample is sequenced using SM-seq.

The work by Tickle et al., 2013, outlined various strategies through which one may generate the informative subsample from the 16S rRNA sequences [9]. They outlined six methods of sample selection, which made use of dissimilarity metrics to attain samples which maximize certain properties of the microbial composition through ranking or clustering. Two of the methods are supervised and thus incorporate a phenotypic label into how the subsample is generated [5]. The work by Tickle et al., made exclusive use of Operational Taxonomic Units (OTUs), and the progress towards the use of Amplicon Sequencing Variants (ASVs) in place of OTUs is the main motivation for this work.

Existing research utilize strategies to select the subsample based on biological diversity and dissimilarity metrics calculated using OTUs [9]. OTUs are generated from sequencing data by clustering sequences which are at least 97% similar to each other. Through clustering of similar sequences, the OTU approach aims to minimize the effect of possible sequencing errors. In contrast, the ASV approach utilizes an error model and all the reads in the sample to generate a probability that any sequence present in the sample was generated as a result of a sequencing error, with only sequences with a probability past a determined threshold being retained as ASVs [17].

The ASV approach has also been shown to exhibit equal or greater specificity and sensitivity to the OTU method [4, 18]. There are various trade-offs and advantages in the use of ASVs compared to OTUs, such as the clustering of sequences at an arbitrary 97% similarity threshold which prohibits the use of OTUs in resolving fine-scale variation [19], and ASVs being exact sequences allowing for ASV results to be readily comparable between studies where the sequences originate from the same target region [17]. Furthermore, a given target gene sequence always generates the same ASV, therefore, an ASV may be compared to a reference database at much higher resolution than an OTU, which allows for precise species identification to the species and sub-species level [17].

It remains unknown if the previously described subsampling strategies are comparable between these two taxonomic qualifications. The effectiveness of data driven strategies for subsample selection is also unclear, as current approaches are primarily biologically driven [9].

The primary goal of this manuscript is to assess whether the subsampling strategies for two-phase metagenomic studies proposed by Tickle et al., that typically utilize OTUs, will lead to different results when using ASVs. We also propose data driven strategies for subsample selection through dimension reduction techniques, as the techniques proposed by Tickle et al. are all biologically driven methods. Lastly, we will evaluate the effects that selecting a different subsample, due to the using ASVs or OTUs, has on the phase-two analysis using shotgun metagenomic sequencing from the selected samples.

## Materials and methods

### Data set

**Fig 1:**
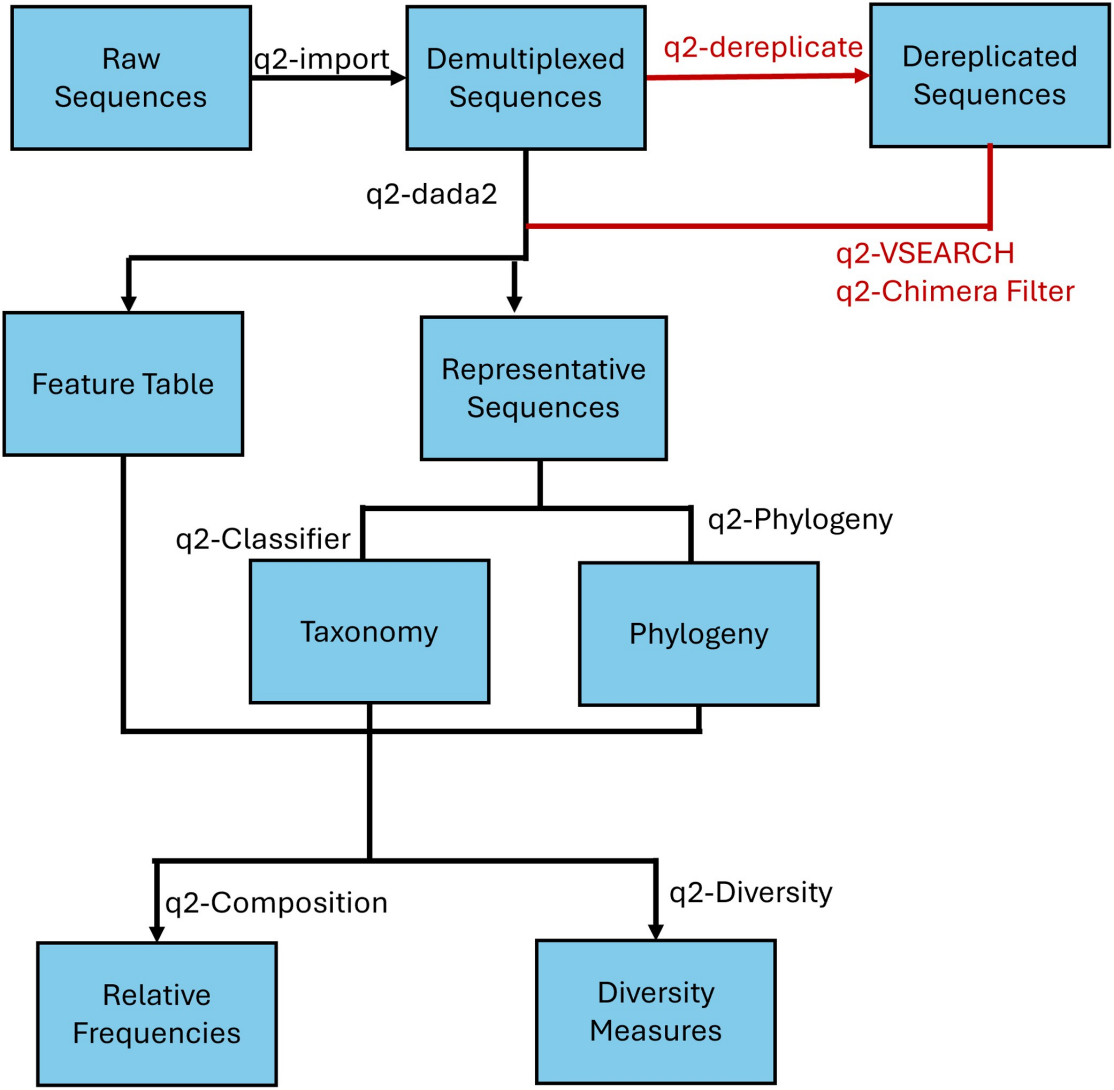
QIIME 2 workflow. Workflow diagram depicting the QIIME 2 workflow. Raw sequences are demultiplexed and processed into feature tables and representative sequences. The steps only done for OTU processing are shown in red. Quality control steps, such as truncating and chimera filtering were performed as outlined in the QIIME 2 documentation. The representative sequences are used to identify the taxonomy and phylogeny, which is used in conjunction with the feature table to generate diversity measures and the relative frequency table.

We accessed 3,349 samples (16S rRNA and/or SM-seq) from the DIABIMMUNE microbiome project (comprising of three cohort studies investigating the gut microbiota of infants) [20] alongside the subject metadata. The sample inclusion criteria was as follows: The sample selected was from the three-country cohort, as it had the highest number of patients and would prevent differences in study populations from affecting the results; The sample collected had both 16S rRNA and SM-seq data measured, so that it could be used in both phases of the study; Only one sample per participant was included in the study, to exclude a temporal aspect to the analysis and prevent any bias based on some subjects being sampled more times than others. These criteria yielded a total of 199 samples of 16S rRNA V4 region and SM-seq. We utilized the Quantitative Insights Into Microbial Ecology 2 (QIIME 2) software version 2021.11 and suggested pipeline to process the raw sequences, as shown in Fig 1.

### OTU processing

We used QIIME 2 [21] to process the demultiplexed 16S rRNA gene amplicon sequence data of the selected subsamples into OTUs using the microbiome data analysis software clustering plugin VSEARCH (via q2-vsearch) [22]. The demultiplexed sequences were first filtered, dereplicated, and then underwent close-clustering OTU generation. Only sequences which shared an 97% or higher similarity sequences in the Silva reference database [23] were retained as OTUs, chimeric sequences were then filtered out resulting in about 27,323 OTUs. Taxonomy data was then generated from the OTUs using the silva-132–99-v3v4-q2–2019 build database of 16S rRNA sequence data, and used to collapse them into 501 OTUs of identical taxonomy.

### ASV processing

We used QIIME 2 [21] to process the demultiplexed 16S rRNA gene amplicon sequence data of the selected subsamples into ASVs using the microbiome data analysis software denoising plugin DADA2 (via q2-dada2) [24]. After visual inspection of quality scores, a truncation length of 150 base pairs was chosen to ensure the quality of the reads retained. During ASV generation, sequences which had an expected error greater than 2.0 were removed, and chimera sequences were detected and removed using the consensus method, resulting in 326 different ASVs [21]. Taxonomy data was then generated for the ASVs using the silva-132–99-v3v4-q2–2019 build database of 16S rRNA sequence data. We also used utilized Deblur (via q2-deblur) [25], a denoising plugin, to contrast two ASV generating methods. As per the Deblur pipeline, the demultiplxed reads were filtered based on their quality score, then ASV generation proceeded using Deblur instead of DADA2 for ASV generation, resulting in 287 ASVs.

### Biologically driven subsampling methods

Relative frequency tables were generated for both ASVs and OTUs by dividing the count of a clade in each sample by the total counts of all clades in that sample. We then used the computational tool microPITA (Huttenhower Lab) [9] on the relative frequency tables in order to determine the biologically driven subsamples using five different methods: representative, extreme, diverse, discriminant, and distinct. Representative, diverse, and extreme subsampling are unsupervised methods, whereas discriminant and distinct subsampling require the use of an additional phenotypic trait of interest. In our case, as the three country cohort aimed to test the hygiene hypothesis, of the metadata available the presence of known allergy was chosen as the phenotype of interest. In the case of subjects with missing allergy information, they were assumed to have no known allergies.

All 5 methods employed one of two widely used biodiversity metrics, the Bray-Curtis (BC) dissimilarity or the Simpson alpha diversity index. Biodiversity metrics measure microbiome diversity within a sample (alpha diversity) or the dissimilarity between two samples (beta diversity).

The BC dissimilarity that was used to quantify the beta diversity between sample *i* and sample *j* is given by the following equation:

$$\begin{array}{c} BC_{ij}=1-\frac{2C_{ij}}{S_{i}+S_{j}} \end{array}$$ (1)

Where *C_ij_* refers to the lesser within-sample sum of the count of species that were common to both sample *i* and sample *j*. *S_i_* and *S_j_* are the sum of all species within sample *i* and *j* respectively.

The Simpson alpha-diversity of a sample is given by the formula:

$$\begin{array}{c} D_{i}=1-(\frac{\sum k(k-1)}{K(k-1)}) \end{array}$$ (2)

Where *D* is the diversity index of *i*, *k* is the total number of organisms of a particular species and *K* is the total number of organisms of all species in the sample.

The five subsample selection methods are defined as follows:

**Representative subsampling**: The BC dissimilarity matrix of the samples was used to perform k-medoid clustering, where the number of clusters required was equal to the *k* samples selected. The samples nearest to the center of each cluster are selected to represent the cluster in the subsample.

**Diverse subsampling**: Samples are ranked based on decreasing inverse of the Simpsons alpha-diversity Index, then the top *k* samples are selected as the subsample.

**Extreme Subsampling**: A sample dissimilarity matrix is constructed using the additive inverse of the BC dissimilarity matrix. Agglomerative hierarchical clustering is then used to build a dendrogram of sample relationships based on the dissimilarity matrix; the *k* most terminal nodes to be joined to the cluster are selected for the subsample.

**Discriminant selection**: Conditional on the phenotype of interest, BC dissimilarities are calculated from the average sample (centroid) of all other classes. As our phenotype of interest is dichotomous, the distance is calculated to the centroid of the opposite class for each sample. The *k* samples with the smallest distances are selected in ascending order.

**Distinct selection**: Similar to discriminant selection, conditionally on the allergy phenotype of a sample, the BC dissimilarities are calculated to the centroid of the opposite class for each sample. The *k* samples with the largest distances are selected in decreasing order.

### Data driven subsampling methods

We employed two dimension reduction techniques as the proposed data driven subsampling methodology. Principal Component Analysis (PCA), which aims to produce linear combinations of the initial variables in such a way that the derived variables capture as much variance as possible [26]. However, as each principal component is a linear combination of all variables in the dataset, they can be difficult to interpret. For this reason we also employed Sparse Principal Component Analysis (SPCA). SPCA is a modified version of PCA which makes use of sparse loadings. Thus the linear combination found by the algorithm is derived from a subset of the initial variables [26].

**Fig 2:**
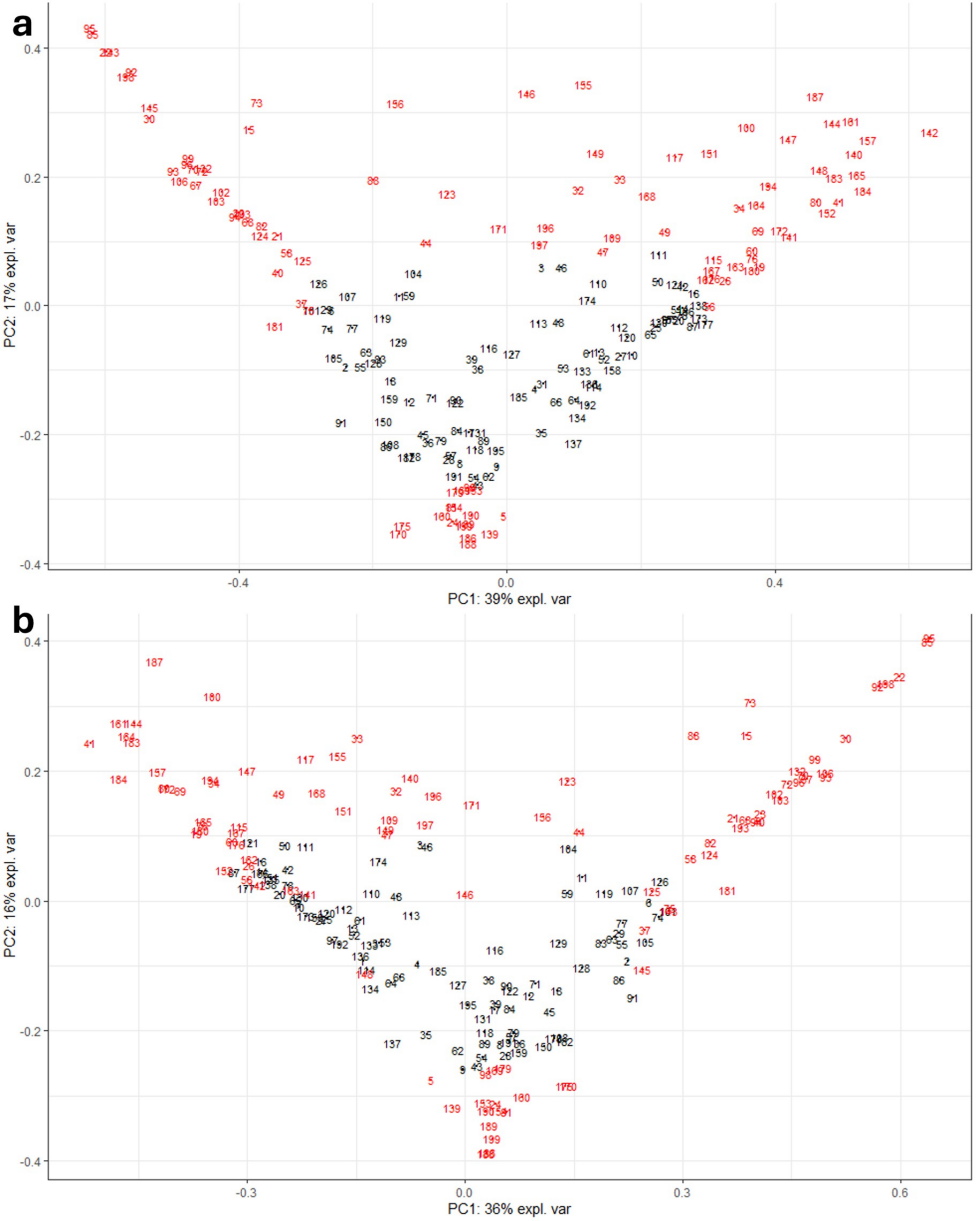
PCA plots of ASV and OTU samples. PCA results at n = 100 for a) ASVs with the first two principal components explaining 56% of the variance in the sample. b) OTUs with the first two principal components explaining 52% of the variance in the sample. The numbers correspond to the alphabetically sorted Sample IDs. The PCA subsample was selected from the extremes of this plot.

The samples were enumerated from lowest to highest sample ID, and PCA was performed based on the variance-covariance matrix using the mixOmics R package [27]. Fig 2 displays how the PCA samples were ordered by the first two principle components which explained over 50% of the variance in both ASV and OTU samples. Subsample selection was then performed as follows: The sample IDs were sorted according to the first principal component; one third of the subsample was selected from each extreme of the first principal component; all non-selected samples were sorted by the second principal component; one sixth of the final subsample was selected from each extreme of the second principal component. Likewise, apart from the creation of the sparse principal components, the subsampling process was the same for the SPCA method (S1 Fig).

### Data analysis

We used the Singularity software (version 3.0.3) [28] on the Scinet Niagara cluster to run QIIME 2 and a virtual conda environment to run Metaphlan 3.0. R version 4.1.2 (2021–11-01) was used for processing the metadata, adjustment of data formatting between software.

The Micropita tool was supplied with the relative frequency tables which then extracted the necessary alpha and beta diversity information to perform the biologically driven subsampling at the n = 20, n = 50, and n = 100 levels.

We used LEfSe [29] to determine the differential representation of taxa between the selected and unselected samples for each subsampling method. LEfSe uses the non-parametric factorial Kruskal-Wallis sum-rank test at an alpha level of 0.05 to detect features with significant differential abundance with respect to subsample selection status [29].

We used the microbial community profiling tool Metaphlan to process and analyse the SM-seq data of the 199 samples. We then merged the processed profiles of the samples that were selected to generate relative frequency tables of the SM-seq data for each subsample methodology at each of the chosen sample sizes.

Lastly we performed LEfSe analysis on each of the profiles using presence of allergy as the comparative class.

## Results

11230477 demultiplexed sequences underwent OTU processing, resulting in 9689039 non chimeric sequences post clustering. This resulted in 27323 OTUs which underwent taxonomic collapse into 501 OTUs. In comparison, after ASV processing a total of 700564 of the input sequences were denoised and considered non-chimeric. This resulted in 2281 individual ASVs which and underwent taxonomic collapsed into 326 ASVs.

**Fig 3:**
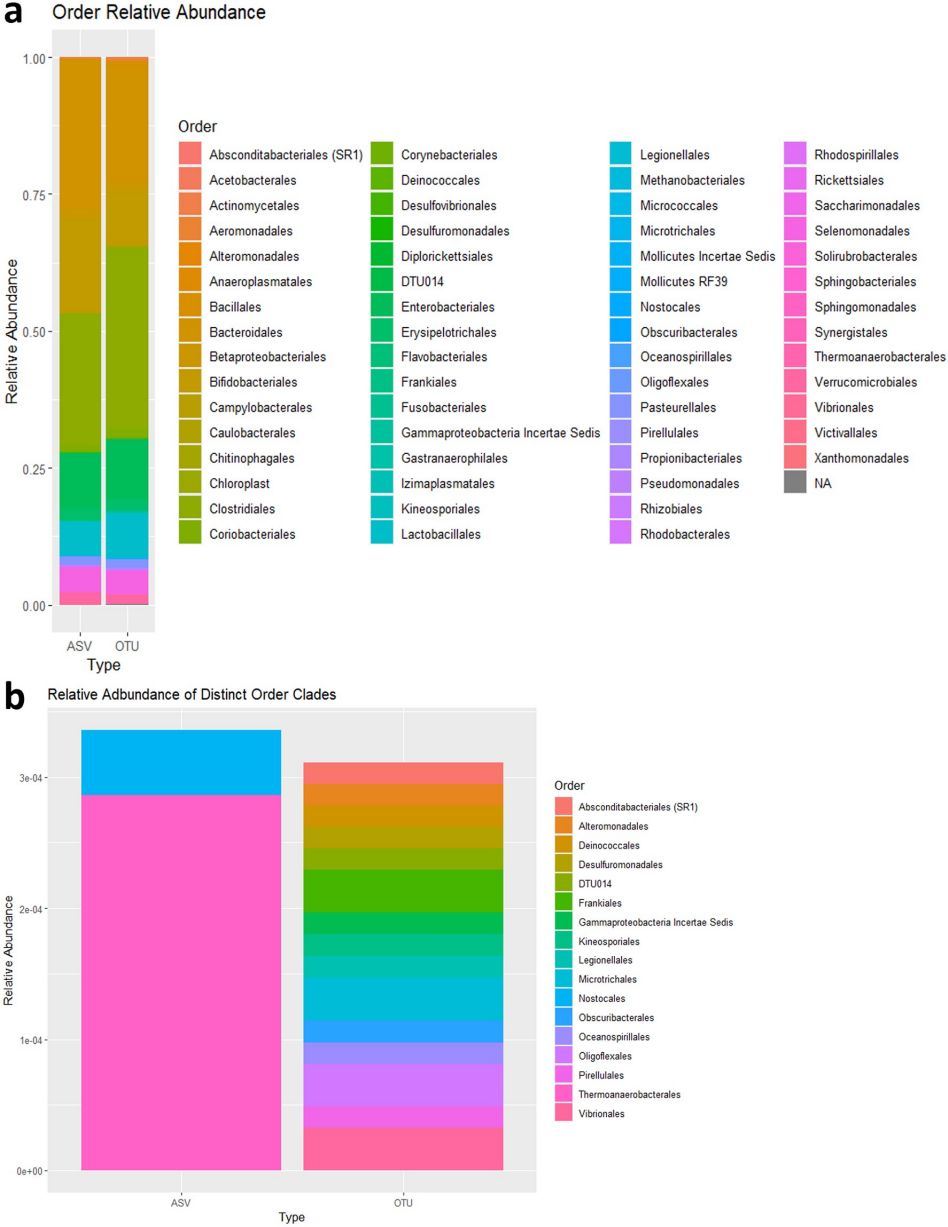
Relative abundance plots of ASVs and OTUs. Barplots showing the relative abundance at the Order clades for both the ASV and OTU tables. a) Barplot comparing the entire order clade of both ASV and OTU samples, showcasing the differences in relative abundances between the overall samples. Of note, some clades in orange are more abundant for ASVs, whereas there are more abundant green clades for OTUs. b) Barplot showing the clades which were uniquely present in each sample.

Both relative frequency tables had similar relative abundances, as shown in Fig 3. The first plot shows the 62 Order level clades found in the samples, and does highlight some differences in the relative abundances. The second plot shows that 2 of the Order clades present in the ASV samples are not present in the OTU samples, where as 15 Order level clades found in the OTU samples were not present in the ASV samples.

Post taxonomic collapse, the ASV table had a total of 326 ASVs, each sample having an average Shannon alpha diversity of 1.89 (+/-0.65), where as the OTU table had 501 OTUs with each sample having an average Shannon alpha diversity of 1.62 (+/-0.68).

**Fig 4:**
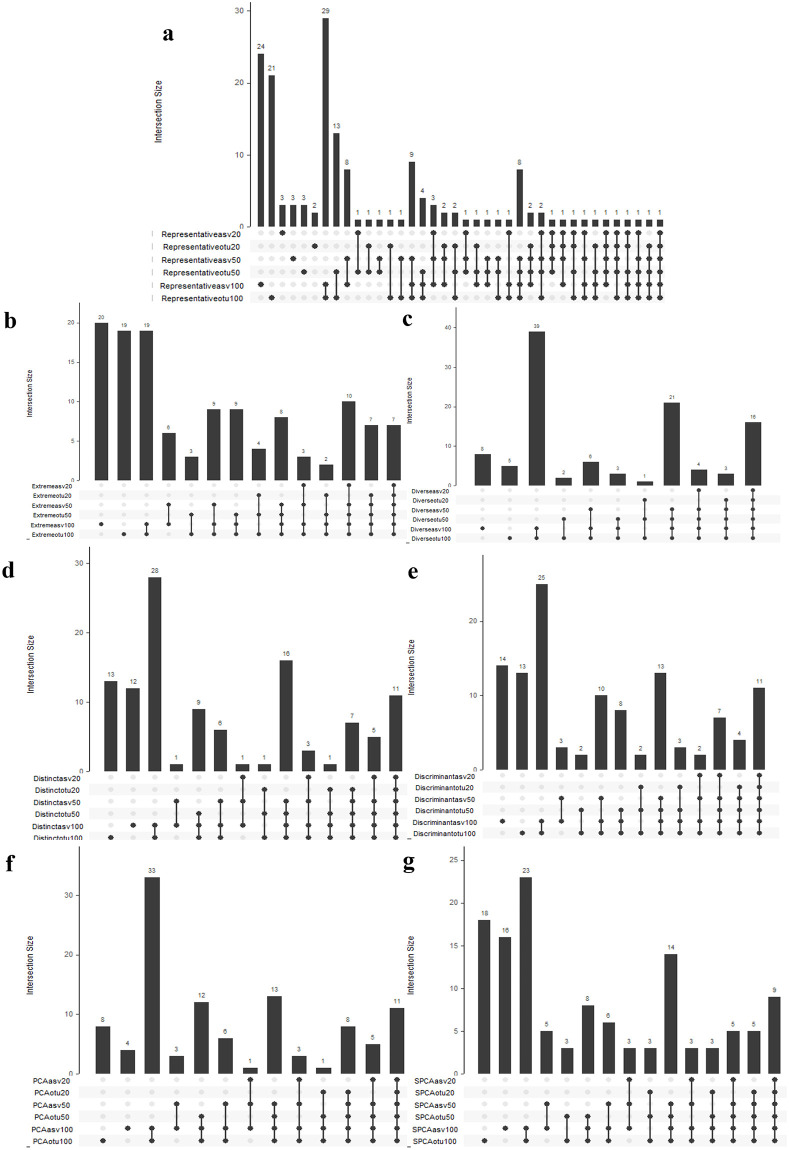
UpSet plot of subsampling with ASV vs OTU. UpSet plots showing agreement between ASV and OTU for each of the selection methods when used to select 10%, 25%, and 50% of the sample using ASVs vs OTUs. a) Representative subsampling, b) Extreme subsampling, c) Diverse subsampling, d) Distinct subsampling, e) Discriminant subsampling, f) PCA subsampling, g) SPCA subsampling. Each column represents the size of a particular set (the singular dots) or the intersection of the sets (represented by the dots connected by a line) containing the same samples. The number of samples within each group appear above the column.

We compared the selected samples by each subsampling strategy when ASVs were used for data processing to when OTUs were used instead at three different sample sizes (20, 50, 100). For ease of comparison, we chose to visually represent these results using UpSet plots (Fig 4). For the majority of the subsampling strategies, there is large overlap (80% or higher), between ASVs and OTUs as to which samples were to be included in the subsamples across sample sizes. This shows that the effectiveness of these subsampling techniques will be preserved when shifting from the use of OTUs to the use of ASVs.

However there are three methods that differ, which are extreme, SPCA and representative subsampling. Extreme and SPCA subsampling both display very different subsamples between ASVs and OTUs at low sample sizes (less than 50% overlap), interestingly the overlap was shown to increase as sample size was increased. Representative subsampling showed little to no overlap in sample selection not only between ASVs and OTUs across sample sizes, but also within the same data processing method, with 35% of samples overlapping between ASVs and OTUs at n = 20, 44% at n = 50, and 57% at n = 100.

### Bacterial representation

**Fig 5:**
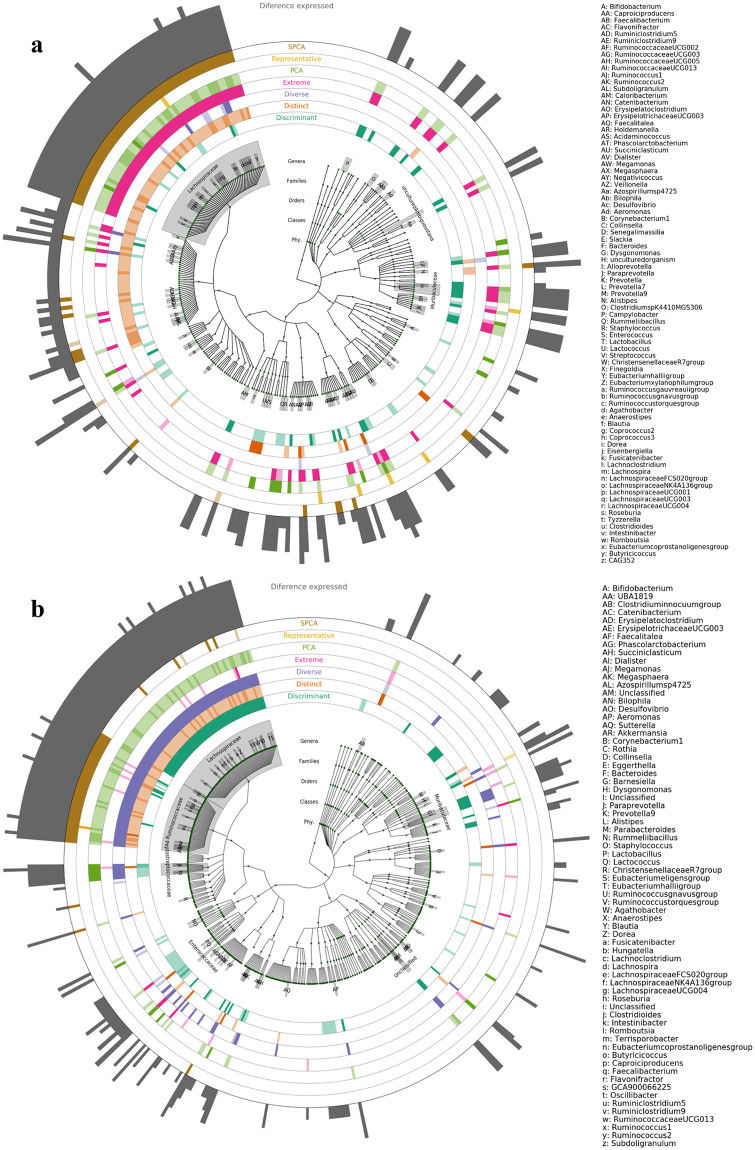
ASV and OTU cladograms. Cladograms of 16S rRNA data showing taxa up to genus level generated using LEfSe. a) Cladogram of ASV subsamples and b) OTU subsamples, both at sample size 100. For each subsampling method, areas highlighted with a dark shade show taxa that are over-represented in the subsample selected by the method used, while areas highlighted with a light shade are taxa which are under-represented in the subsample compared to sample population. The outermost ring is composed of bars showing how many times a clade was selected. Clades that are highlighted by 3 or more methods are highlighted and added to the legend.

We hypothesized that there would be no significant differences in the bacterial representation of subsamples when using ASVs instead of OTUs for each of the methodologies utilized. To test this, we performed LEfSe analysis for each of the methods at three sample sizes, comparing the bacterial composition of the samples included in the subsample to those not included. We were then able to compare the subsamples processed using ASVs to subsamples processed using OTUs for each of the methods. These comparisons showed highly similar bacterial composition between subsamples selected using ASVs compared to using OTUs, regardless of subsample size or sampling methodology. Cladograms depicting the taxonomic relation of clades, were used to identify and highlight key clades that were either over-represented or under-represented in the subsamples in the LEfSe analysis at subsample sizes of 20, 50, and 100 samples (S4 and S5 Figs and Fig 5 respectively).

The cladograms for samples processed using ASVs show highly similar results for each of the subsampling methods utilized as the cladograms samples processed using OTUs at equal sample sizes. The SPCA, PCA, discriminant and diverse subsampling strategies show a trend of highlighting similar clades as each other for both the ASV and OTU cladograms across sample sizes. The highest bars of the outermost ring of the cladograms in Fig 5 show the clades that were identified as over or under represented in six of the seven subsampling methods. These genus clades were *Lachnoclostridium* and *Intestinibacter* for the ASV samples, and *Subdoligranlum*, *Butyricicoccus*, and *Caproiciproducens* for the OTU samples.

We further compared the results between the use of DADA2 and Deblur for generating ASVs. S6–S8 Figs show the LEfSe results for each subsampling method being used on the samples processed by DADA2 and Deblur. These cladograms show that across the board, the taxa highlighted in the Deblur processed samples are highly similar to those highlighted in the DADA2 samples. Furthermore, due to the similarity in results between the OTU processed samples to the ASV (DADA2) samples, this shows that all three 16s rRNA processing methods yield similar subsamples.

### Downstream SM-seq

We processed the SM-seq data of the same 199 individuals using Metaphlan 4.0 in order to assess the downstream effect that utilizing each of the subsampling methods would have on the second phase of a microbial study.

**Fig 6:**
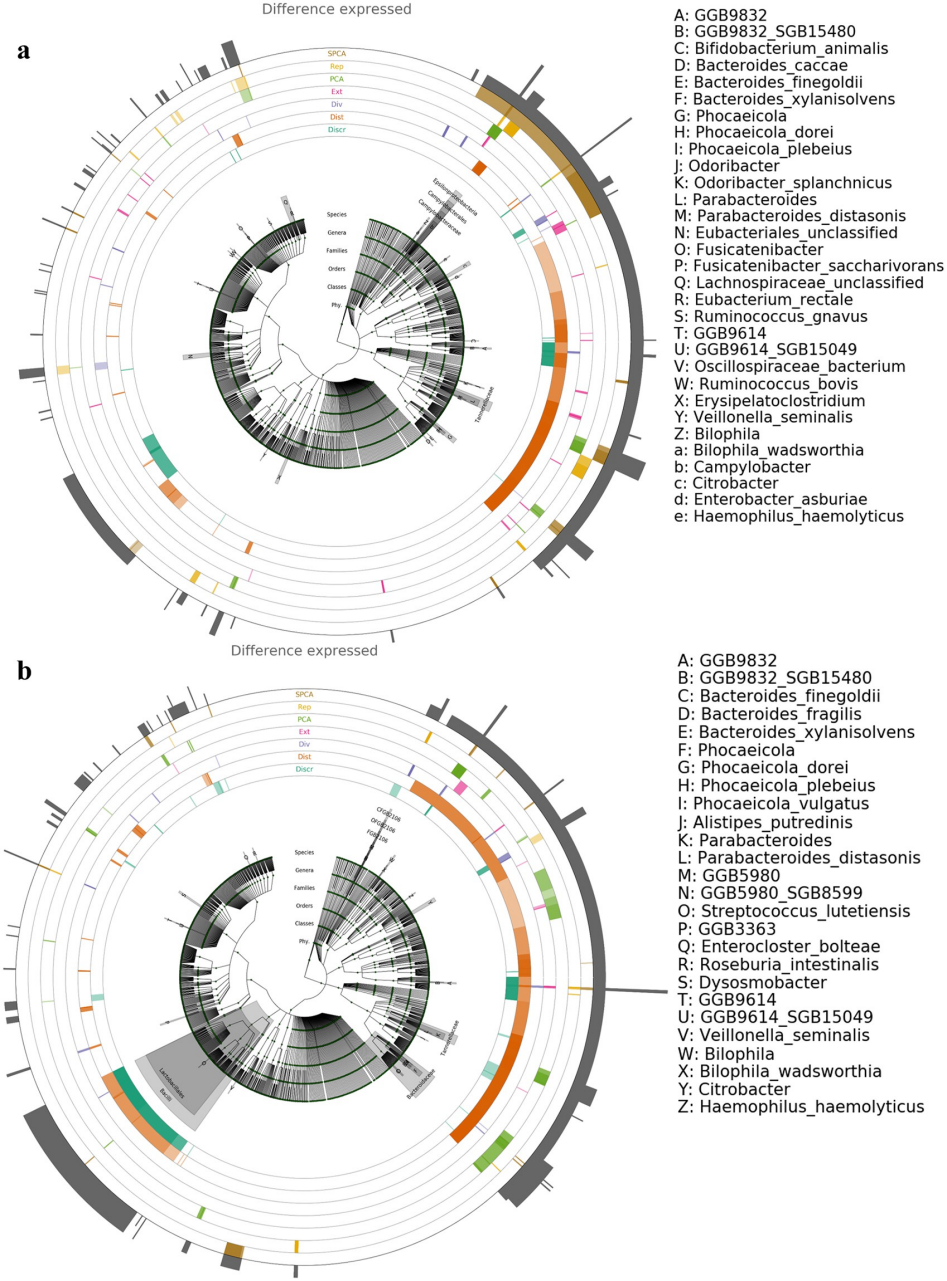
SM-seq cladograms. Cladograms of SM-seq data comparing presence of allergy. a) subsamples selected from ASV processed 16S rRNA, b) subsamples selected from OTU processed 16S rRNA, both at subsamples of n = 100. For each subsampling method, areas highlighted with a dark shade show taxa that are over-represented in the subsample selected by the method used, while areas highlighted with a light shade are taxa which are under-represented according to LeFSe results, which compared allergic and non-allergic individuals within each subsample.

We successfully profiled the SM-seq data into microbial compositions of the 199 samples. Subsets of the metaphlan profiles were generated according to the selected subsamples from phase one and used to generate merged abundance tables. The corresponding SM-seq of the selected subsamples were used for LEfSe analysis to emulate phase two analysis of a two-phase microbial study. For each subsample, the presence of allergy was used as the phenotype of interest on which the microbial compositions were evaluated. These results are shown in Fig 6, which allowed us to compare which microbes were more or less represented in allergic individuals compared to non-allergic individuals across subsamples. The number of clades highlighted is very similar between both Cladograms, with 31 clades being shown to be of interest in 3 or more of the methods for the ASV subsamples, whereas 26 clades were for the OTU subsamples. The results at the other two sample sizes n = 20, and n = 50 are shown in S9 and S10 Figs respectively.

*Bifidobacterium* are consistently shown to be underrepresented in non-allergic individuals across sample sizes in the ASV subsamples. No such pattern was observed in the OTU subsamples. The bottom left sector of the cladogram gained more prominence in over/under represented taxa as the sample size increased. As the sample size increased the number of taxa that had a significant difference between allergic and non-allergic individuals appeared to increase across the different methods for the subsamples derived using both ASVs and OTUs at the 16S rRNA level. Likewise, the number of taxa of interest (taxa highlighted by more than one method) also, increased as the sample size increased. The level of overlap for the taxa of interest between the ASV and OTU subsamples increased as sample size increased from 0 at n = 20, to 2 at n = 50, to 16 at n = 100.

We also compared the subsampling results of DADA2 and Deblur at the SM-seq level. S11–S13 Figs show the LEfSe results comparing allergic and non-allergic individuals in each of the subsamples. As shown in the cladograms, the majority of the subsampling methods had similar LEfSe results. The main exception was extreme subsampling, which highlighted very few taxa at the lowest samples size and increased the number of taxa highlighted as the sample size increased for the Deblur samples, whereas it greatly increased the number of highlighted taxa at the highest sample size for the DADA2 samples. The extreme subsampling method selected almost all the same samples for the n = 20 sample, but the overlap diverged as the sample size increases, which may explain the different results. Aside from extreme subsampling the similarity in results between the OTU processed samples to the ASV (DADA2) samples, shows that all three 16s rRNA processing methods had similar effects on the downstream SM-seq post subsampling.

## Discussion

In this paper we have compared the effects of using ASVs instead of OTUs in two-phase microbial studies that implement a subsampling strategy. Our results indicate that using ASVs for microbiome sequencing in the first-phase of microbial trials usually yields a similar subsample to using OTUs. The abundance table generated through processing the 16S rRNA data using ASVs differs from the table generated using OTUs, in both which microorganisms were identified, and the abundance values themselves. Fig 3 shows that the ASV and OTU samples vary greatly in their relative abundance at the Order level. This may in part be due to the difference in the 16S rRNA processing. The ASV process resulted in much fewer non-chimeric sequences prior to taxa-collapse than the OTU processing, about an order of magnitude lower. This difference was attributed to two main factors, the difference between denoising, that removes erroneous or noisy reads, and closed clustering, which retains all sequences with a 97% similarity to the reference database. The other possible source of this observed disparity in processed sequences is the difference in filtering. The default filtering method used for the OTUs may have been too lenient in comparison to ASVs, where all sequences longer than 150 sequences were filtered out due to low quality scores. Through investigating the effect these differences have in subsample selection and downstream analysis, we determined that ASVs are as efficient as OTUs.

The UpSet plots showed a high level of overlap for the majority of the subsampling methods, suggesting that the structural differences have a minor effect on the extreme and diverse subsampling methods that aim to identify samples which are most-dissimilar to others and those that contain the most within-sample diversity respectively, leading to the high overlap between the ASV and OTU samples selected. In a similar vein, the structural difference between the ASVs and OTUs have minimal effects on the discriminant and distinct subsampling methods, which select samples based on how similar the samples of a particular class are, on average, to the centroids of all other classes. In this case, based on how similar samples from individuals with one allergy status are to those with the opposite allergy status. Overall, switching from OTUs to ASVs seemed to have a minor impact on the diversity and dissimilarity metrics. Furthermore, the high overlap across sample sizes can be attributed to how these methods “rank” all samples and select the n-highest or n-lowest samples. Since increasing sample size has no effect on the “rank” order, it is expected that all samples selected at a smaller sample size are automatically included at higher samples sizes.

The difference in clade generation between ASVs and OTUs would have the greatest impact in the representative subsampling method. The two abundance tables yield structurally different Bray-Curtis matrices, effecting the creation of the n-centroids and the selection of the samples to those centroids. As for the low level of overlap within the same data processing strategy at different sample sizes, this is attributed to centroid generating process, which is highly dependent on the number of centroids required. Hence, there is a low likelihood of centroids being placed in the exact same position at two differing sample sizes, allowing for the same sample to be selected again. The structural differences between the ASV and OTU tables would influence the PCA and SPCA subsampling results in a similar manner. The change in the scores when comparing ASVs and OTUs was not substantial in many of the samples samples, but samples which had greater variance under ASV processing than OTU processing would have a greater PCA scores and thus a higher chance of being selected than previously and vice versa, since the methodology used aimed to select samples from the extremes of the first two principal components. Overall, the structural differences between two relative abundance tables explained the discrepancies shown in the UpSet plots.

After subsample selection we then assessed the effect that the differences between ASV and OTU generation have in the bacterial makeup of the subsample. The center of the cladogram shows the relation between the different taxa identified by the sequencing method used, and it is evident that there are some differences between the cladograms from the ASV processing and those from the OTU processing. The structural differences between the two cladograms, shown by the differing branches in the cladograms, have a significant effect on the subsample selection.

Inspection of the pattern of the clades selected by each subsampling method serves to further exemplify this point. Representative subsampling highlights the fewest clades of all the methods in all instances, as it aims to preserve the microbial representation of the overall sample, hence few clades are shown to be over/under-represented, and this was true across sample sizes for both ASVs and OTUs. The extreme subsampling method aims to select the samples most dissimilar to all other samples, including other samples selected by the method. The fact that the PCA, SPCA, distinct and diverse subsampling methods highlighted many of the same clades in all six cladograms is caused by the shared aim of these methods to select the samples with the most variation.

As far as the key clades highlighted by each methodology, the similarity of the key clades of each subsampling methodology across ASVs and OTUs lends credence to the change to ASVs not impacting the ability of the methods selecting the informative subsample. The LEfSE results repeatedly showed that the differences in microbial composition between selected and unselected samples, were very similar when using either ASVs or OTUs under each of the subsampling methods at each of the sample sizes used. Each of the biologically driven subsampling strategies serve the purpose of identifying a subsample that captures a particular property from the sample population, such as a microbial composition similar to the overall sample or one comprised of the most dissimilar samples. The LEfse analysis results displayed in Fig 6, S6 and S7 Figs show that the selected subsamples appear to contain these properties when using either ASVs or OTUs.

The LEfSe analysis of the representative subsamples highlights few taxa as being over or under abundant when comparing allergic individuals in the subsample to those without allergy. This remains true across sample sizes for both the subsamples. As a representative subsample aims to capture the microbial diversity of the overall sample, it is most akin to what would be observed if the total population could be used as the sample size increases. That few taxa are highlighted by this method gives credence to the taxa being highlighted by the other methods to have risen due to the selection bias introduced by the subsampling methodology.

The result of the LEfSe analysis from the extreme subsampling is expected. As shown in the Fig 4 the ASV and OTU subsamples become much more similar as the sample size increased, leading to nearly identical results for the ASV and OTU subsamples. Furthermore not many clades are selected as being over or under represented in the allergic individuals compared to the non-allergic individuals, likely because the basis of extreme subsampling is the selection of those most dissimilar to other samples, leading to few common patterns being observed between those individuals.

The LEfSe analysis results of diverse subsampling were very similar between the OTU and ASV subsamples, which is expected due to the almost identical subsample selection. Of note is the result for the OTU sample at sample size n = 50, where the *Firmicutes* phylum was found to be overall less present in allergic individuals compared to allergic individuals. This was congruent with the results at the genus level for other subsamples selected using the diverse subsampling method.

The LEfSe analysis results of distinct subsampling highlighted the most taxa for the ASV subsamples at all sample sizes and for the OTU subsamples at n = 100. The results observed with the subsample selected from ASV processed samples is more congruent with expectations, as distinct subsampling selects the non-allergic samples that are most different from the centroid of all allergic individuals and the allergic samples which are most different from the centroid of all non-allergic individuals. Opposite to distinct subsampling, discriminant subsampling highlighted very few taxa across all cladograms. This is also expected, as the non-allergic individuals chosen are the most similar to the centroid of the allergic individuals, and vise-versa.

LEfSe analysis of the PCA subsample selected few taxa as differing in abundance between allergic and non-allergic individuals in both the ASV and OTU subsamples. Furthermore, the number of taxa highlighted in both cladograms is mostly unaffected by the change in sample size. This could perhaps be attributed to the PCA subsampling being made up of the samples with the highest and lowest scores in the first two principal components, making them highly variable in the bacterial abundances leading to the unexpected results at the small sample size.

Compared to PCA, SPCA subsampling had quite different results despite the similar nature of the subsampling methodology. LEfSe analysis showed few results for the ASV and OTU subsamples at the lower sample sizes but highlighted numerous clades at the n = 100 sample size.

The fact that the subsamples highlighted different clades emphasizes that two-phase study making use of a subsampling technique must do so with a clear purpose and objective, otherwise they are introducing unnecessary bias to their results. Furthermore, comparing the cladograms of the same sample sizes based only on the difference introduced by the choice of ASV or OTU showed very similar patterns observed across subsampling methods, which grew more similar as sample size increased.

There were some limitations to our work. The data set that we utilized only contained data of the microbiome of the gut of infants, so in the future we would like to explore the microbiome of other body sites and other age groups. Also, the algorithm we used to generate the subsamples to perform SM-seq could be generalized and streamlined further to make it a robust approach. In this manuscript we compared the use of ASVs to OTUs in generating subsamples for two-phase microbiome studies and found that ASVs are comparable to OTUs. We also found that the data driven strategies we proposed in the manuscript show encouraging performance compared to existing biologically driven strategies used in other works.

## Supporting information

S1 Fig SPCA plots for ASV and OTU data. SPCA plots for the ASV and OTU data at n = 100, processing was similar to PCA, and show similar results. Sub samples were selected using similar methodology as for PCA using the SPCA results.
(TIF)

S2 Fig Scree plots of PCA for ASV and OTU data. Scree plots for the ASV and OTU data showing the variance expressed by the first 7 principal components. Cumulatively these principal components explained –% for ASVs and % for OTUs of the variance found in the samples.
(TIF)

S3 Fig Scree plots of SPCA for ASV and OTU data. Scree plots for the ASV and OTU data showing the variance expressed by the first 7 principal components. Cumulatively these principal components explained –% for ASVs and % for OTUs of the variance found in the samples.
(TIF)

S4 Fig ASV and OTU cladograms. Cladograms of 16S rRNA data showing the clades more or less represented in the subsamples compared to those not selected. a) subsamples selected from ASV processed 16S rRNA, b) subsamples selected from OTU processed 16S rRNA, both at subsamples of n = 20.
(TIF)

S5 Fig ASV and OTU cladograms. Cladograms of 16S rRNA data showing the clades more or less represented in the subsamples compared to those not selected. a) subsamples selected from ASV processed 16S rRNA compared to unselected samples, b) subsamples selected from OTU processed 16S rRNA compared to unselected samples, both at subsamples of n = 50.
(TIF)

S6 Fig DADA2 and Deblur cladograms. Cladograms of 16S rRNA data showing the clades more or less represented in the subsamples compared to those not selected. a) subsamples selected from DADA2 processed 16S rRNA compared to unselected samples, b) subsamples selected from Deblur processed 16S rRNA compared to unselected samples, both at subsamples of n = 20.
(TIF)

S7 Fig DADA2 and Deblur cladograms. Cladograms of 16S rRNA data showing the clades more or less represented in the subsamples compared to those not selected. a) subsamples selected from DADA2 processed 16S rRNA compared to unselected samples, b) subsamples selected from Deblur processed 16S rRNA compared to unselected samples, both at subsamples of n = 50.
(TIF)

S8 Fig DADA2 and Deblur cladograms. Cladograms of 16S rRNA data showing the clades more or less represented in the subsamples compared to those not selected. a) subsamples selected from DADA2 processed 16S rRNA compared to unselected samples, b) subsamples selected from Deblur processed 16S rRNA compared to unselected samples, both at subsamples of n = 20.
(TIF)

S9 Fig SM-Seq cladograms. Cladograms of SM-seq data comparing presence of allergy. a) subsamples selected from ASV processed 16S rRNA, b) subsamples selected from OTU processed 16S rRNA, both at subsamples of n = 20.
(TIF)

S10 Fig SM-Seq cladograms. Cladograms of SM-seq data comparing presence of allergy. a) subsamples selected from ASV processed 16S rRNA, b) subsamples selected from OTU processed 16S rRNA, both at subsamples of n = 50.
(TIF)

S11 Fig SM-Seq cladograms. Cladograms of SM-seq data comparing presence of allergy. a) subsamples selected from DADA2 processed 16S rRNA, b) subsamples selected from Deblur processed 16S rRNA, both at subsamples of n = 20.
(TIF)

S12 Fig SM-Seq cladograms. Cladograms of SM-seq data comparing presence of allergy. a) subsamples selected from DADA2 processed 16S rRNA, b) subsamples selected from Deblur processed 16S rRNA, both at subsamples of n = 50.
(TIF)

S13 Fig SM-Seq cladograms. Cladograms of SM-seq data comparing presence of allergy. a) subsamples selected from DADA2 processed 16S rRNA, b) subsamples selected from Deblur processed 16S rRNA, both at subsamples of n = 100.
(TIF)
