# Supplementary figures and images for: Comparing subsampling strategies for metagenomic analysis in microbial studies using amplicon sequence variants versus operational taxonomic units
Source: PLoS One. 2024 Dec 30;19(12):e0315720. doi: 10.1371/journal.pone.0315720 (PMC11684612; doi:10.1371/journal.pone.0315720)

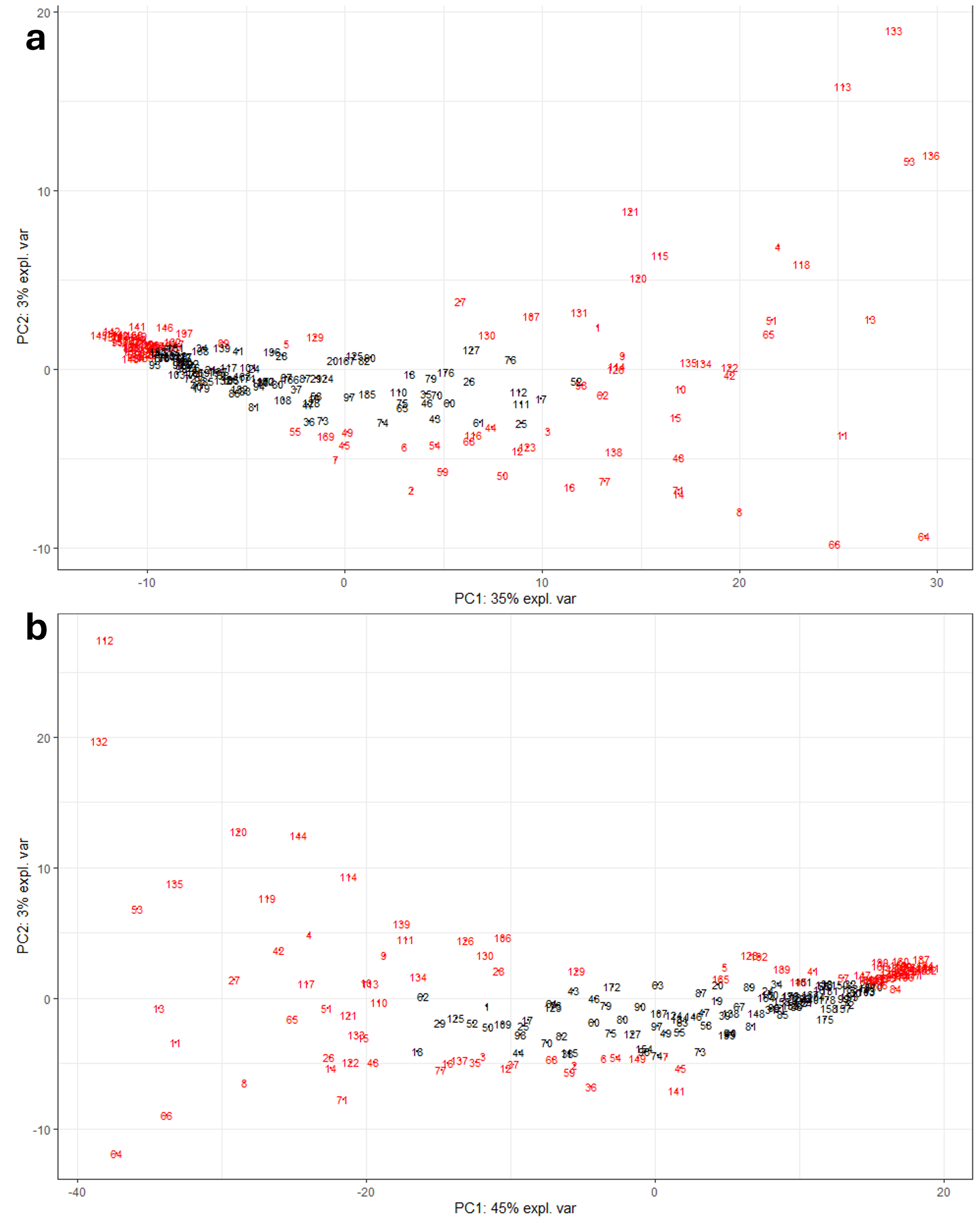

Supplement: S1 Fig — SPCA plots for the ASV and OTU data at n = 100, processing was similar to PCA, and show similar results. Sub samples were selected using similar methodology as for PCA using the SPCA results. (TIF) [file pone.0315720.s001.tif]

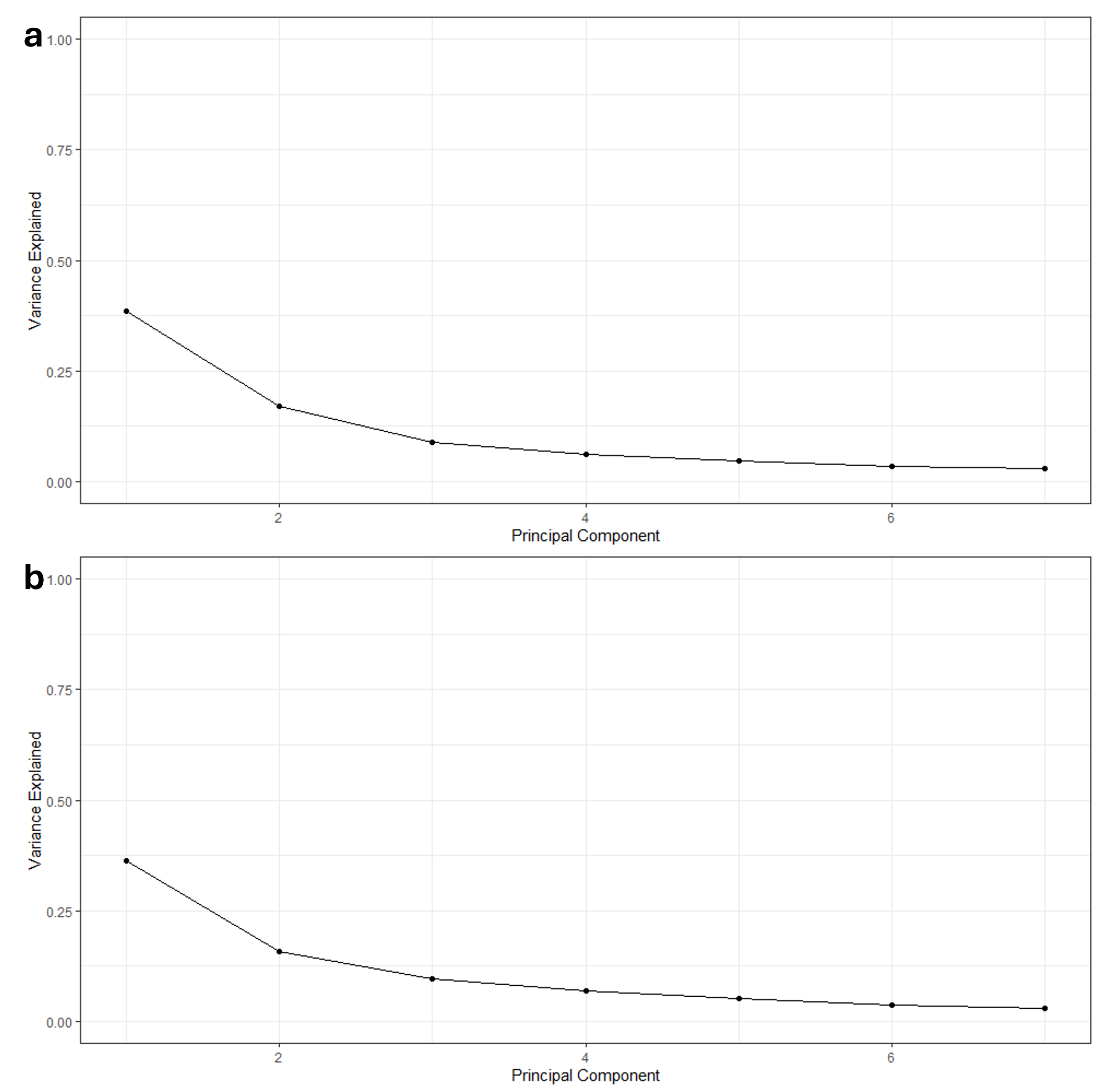

Supplement: S2 Fig — Scree plots for the ASV and OTU data showing the variance expressed by the first 7 principal components. Cumulatively these principal components explained –% for ASVs and % for OTUs of the variance found in the samples. (TIF) [file pone.0315720.s002.tif]

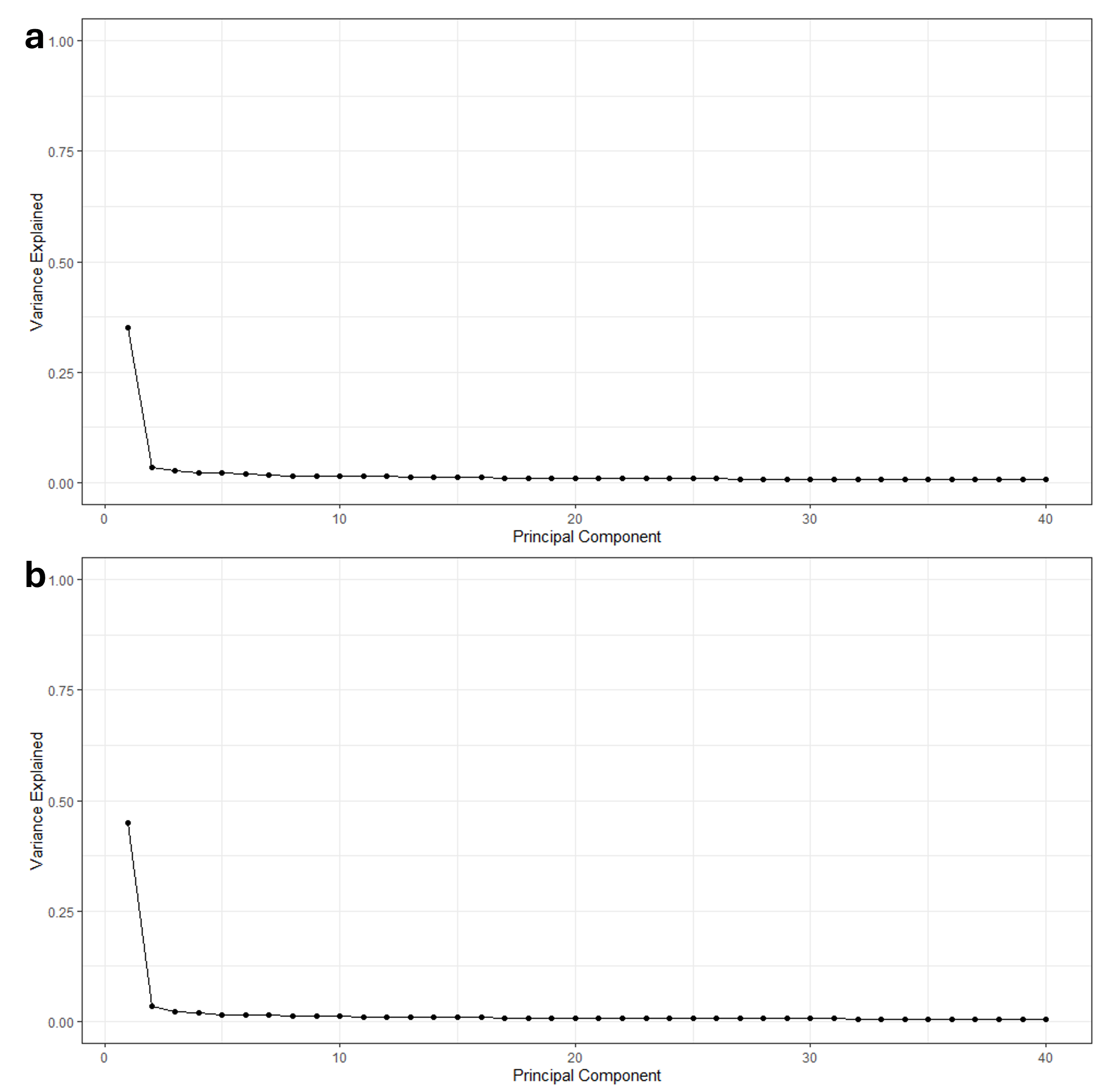

Supplement: S3 Fig — Scree plots for the ASV and OTU data showing the variance expressed by the first 7 principal components. Cumulatively these principal components explained –% for ASVs and % for OTUs of the variance found in the samples. (TIF) [file pone.0315720.s003.tif]

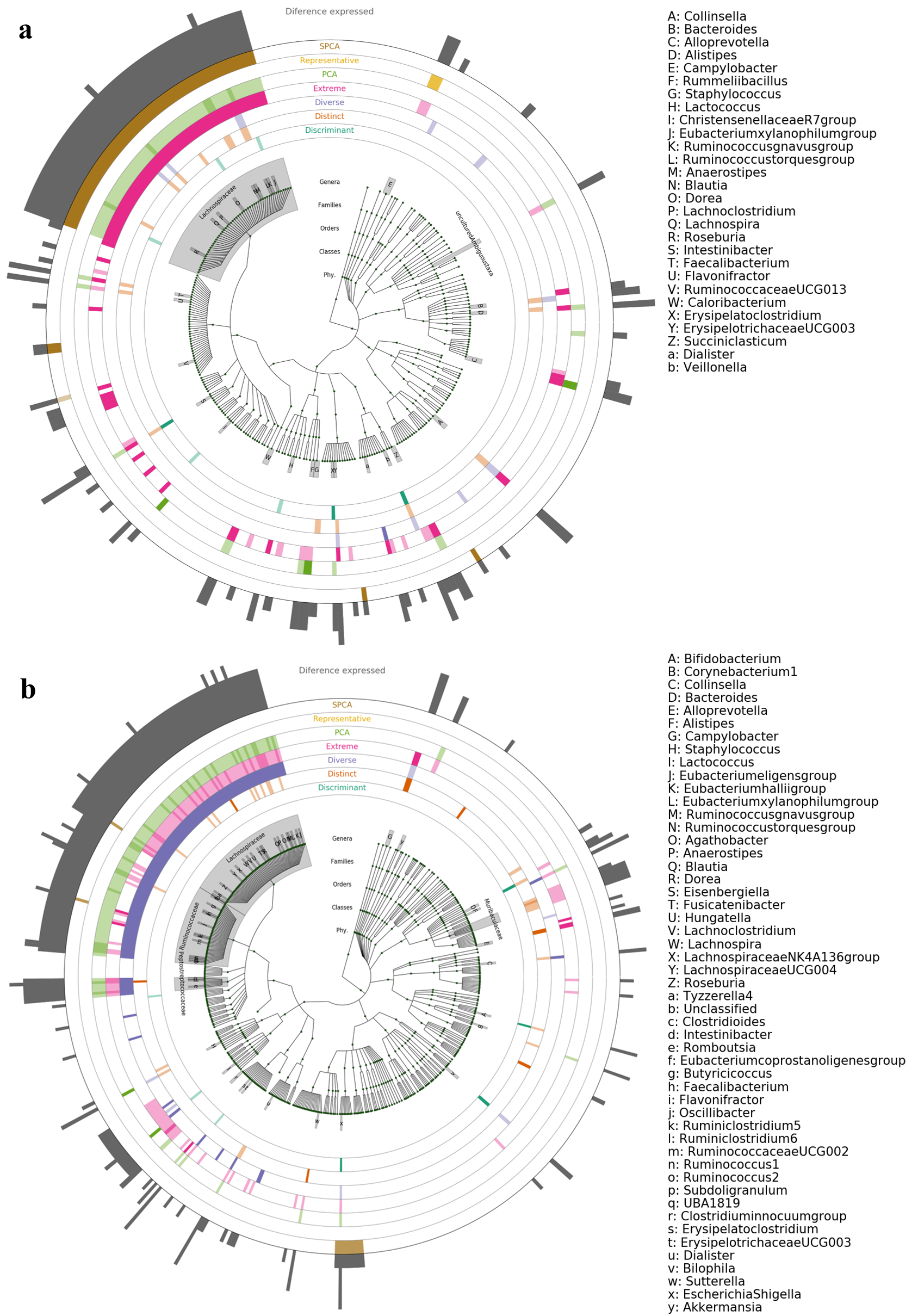

Supplement: S4 Fig — Cladograms of 16S rRNA data showing the clades more or less represented in the subsamples compared to those not selected. a) subsamples selected from ASV processed 16S rRNA, b) subsamples selected from OTU processed 16S rRNA, both at subsamples of n = 20. (TIF) [file pone.0315720.s004.tif]

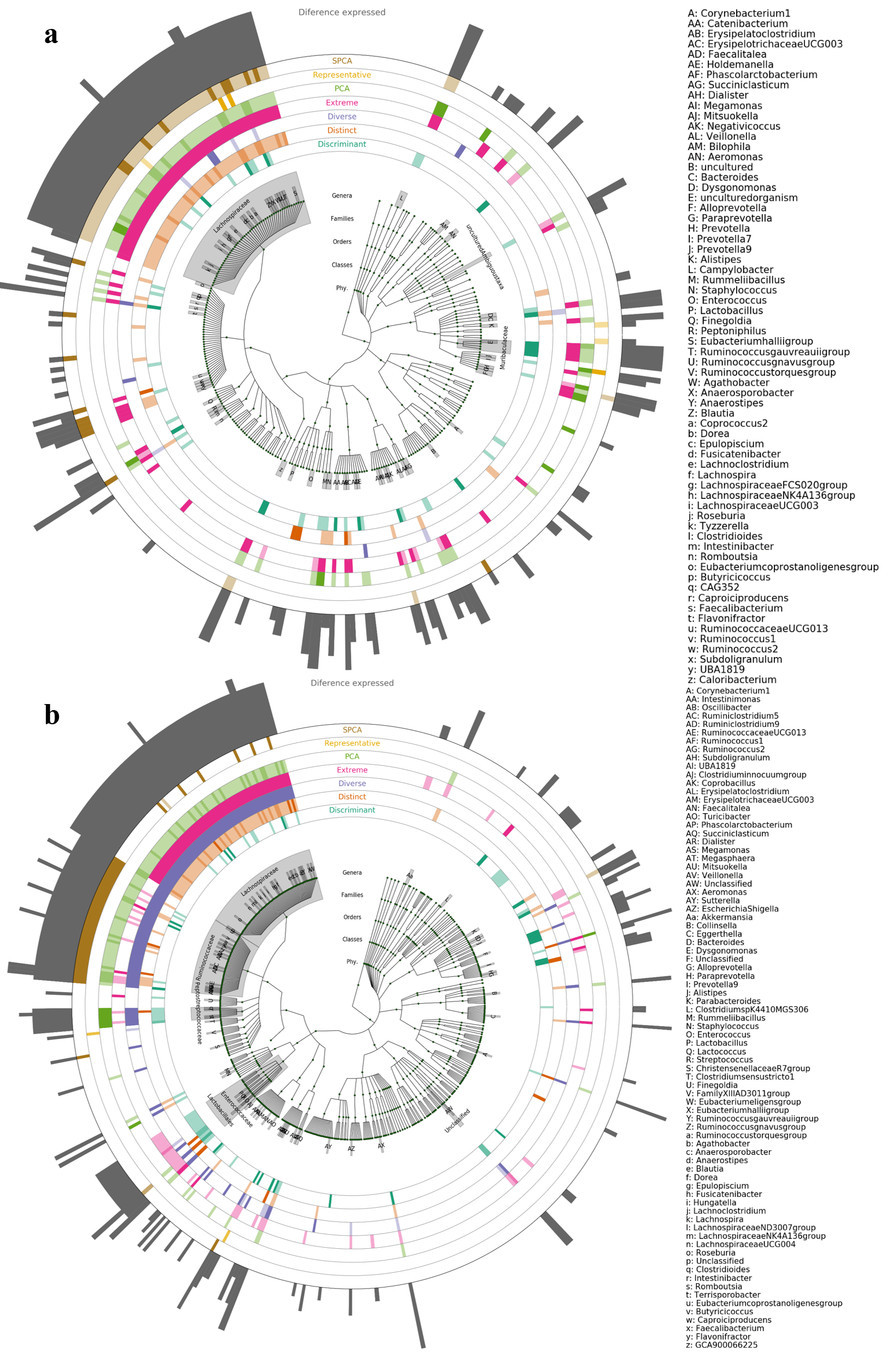

Supplement: S5 Fig — Cladograms of 16S rRNA data showing the clades more or less represented in the subsamples compared to those not selected. a) subsamples selected from ASV processed 16S rRNA compared to unselected samples, b) subsamples selected from OTU processed 16S rRNA compared to unselected samples, both at subsamples of n = 50. (TIF) [file pone.0315720.s005.tif]

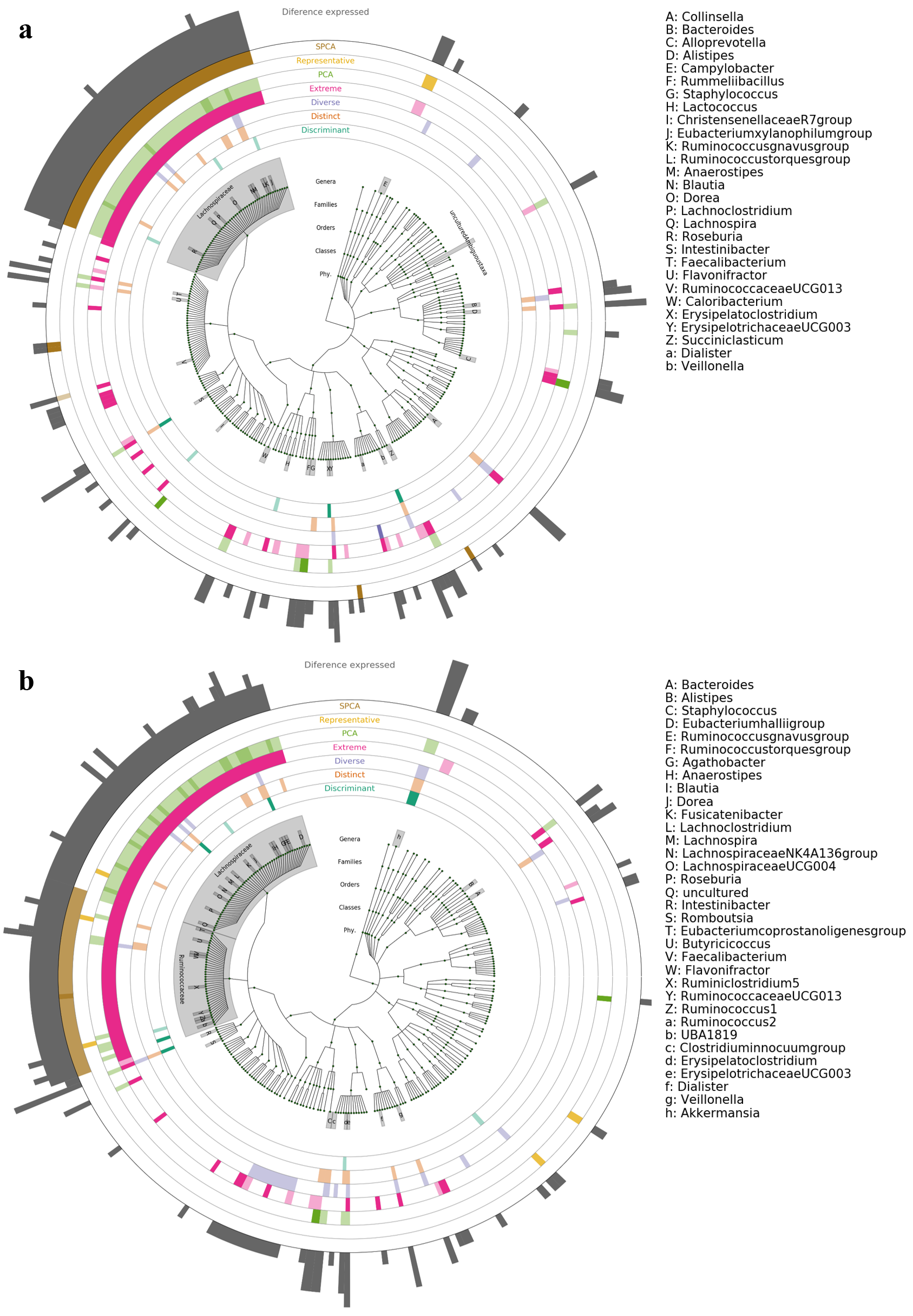

Supplement: S6 Fig — Cladograms of 16S rRNA data showing the clades more or less represented in the subsamples compared to those not selected. a) subsamples selected from DADA2 processed 16S rRNA compared to unselected samples, b) subsamples selected from Deblur processed 16S rRNA compared to unselected samples, both at subsamples of n = 20. (TIF) [file pone.0315720.s006.tif]

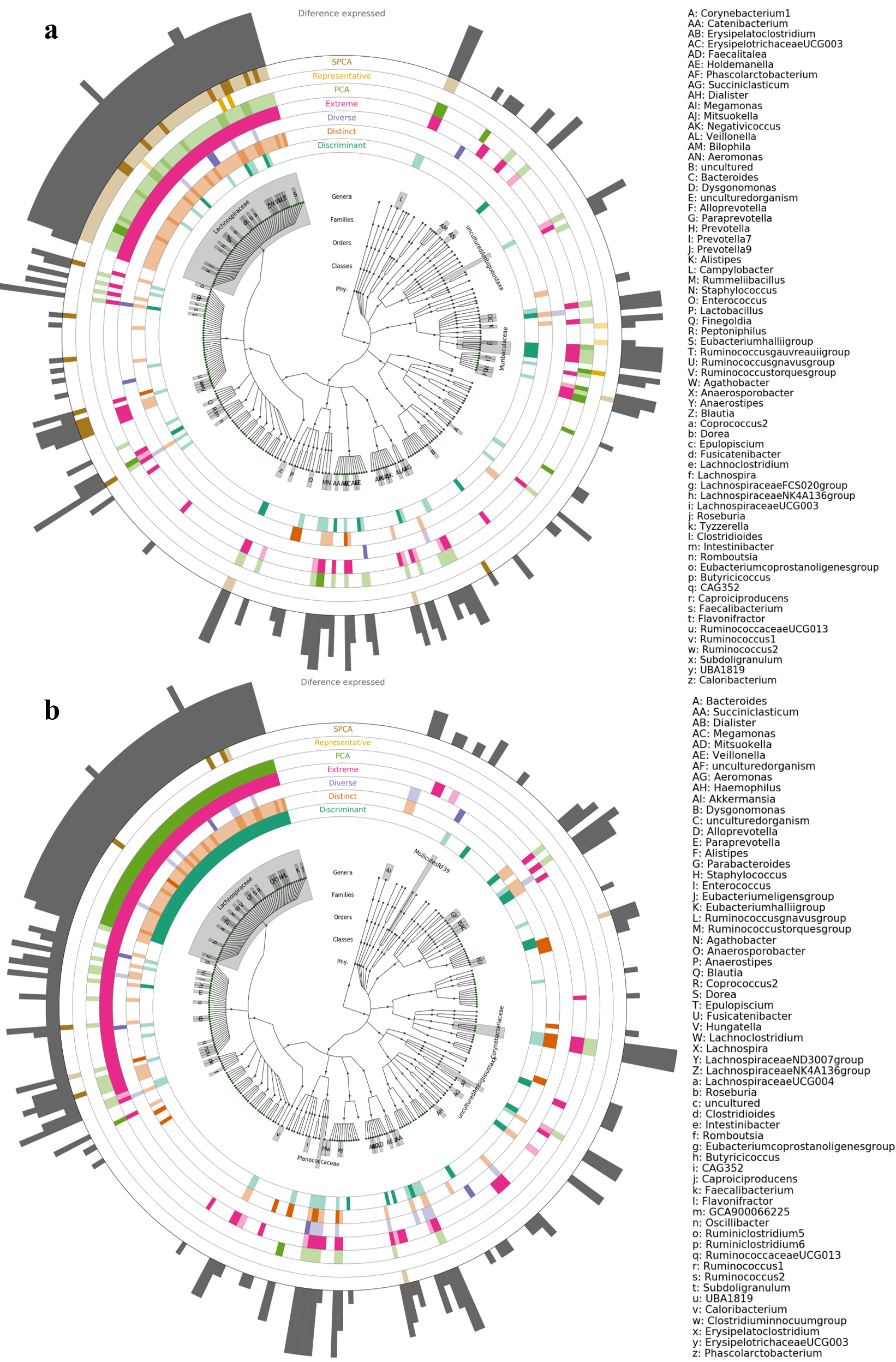

Supplement: S7 Fig — Cladograms of 16S rRNA data showing the clades more or less represented in the subsamples compared to those not selected. a) subsamples selected from DADA2 processed 16S rRNA compared to unselected samples, b) subsamples selected from Deblur processed 16S rRNA compared to unselected samples, both at subsamples of n = 50. (TIF) [file pone.0315720.s007.tif]

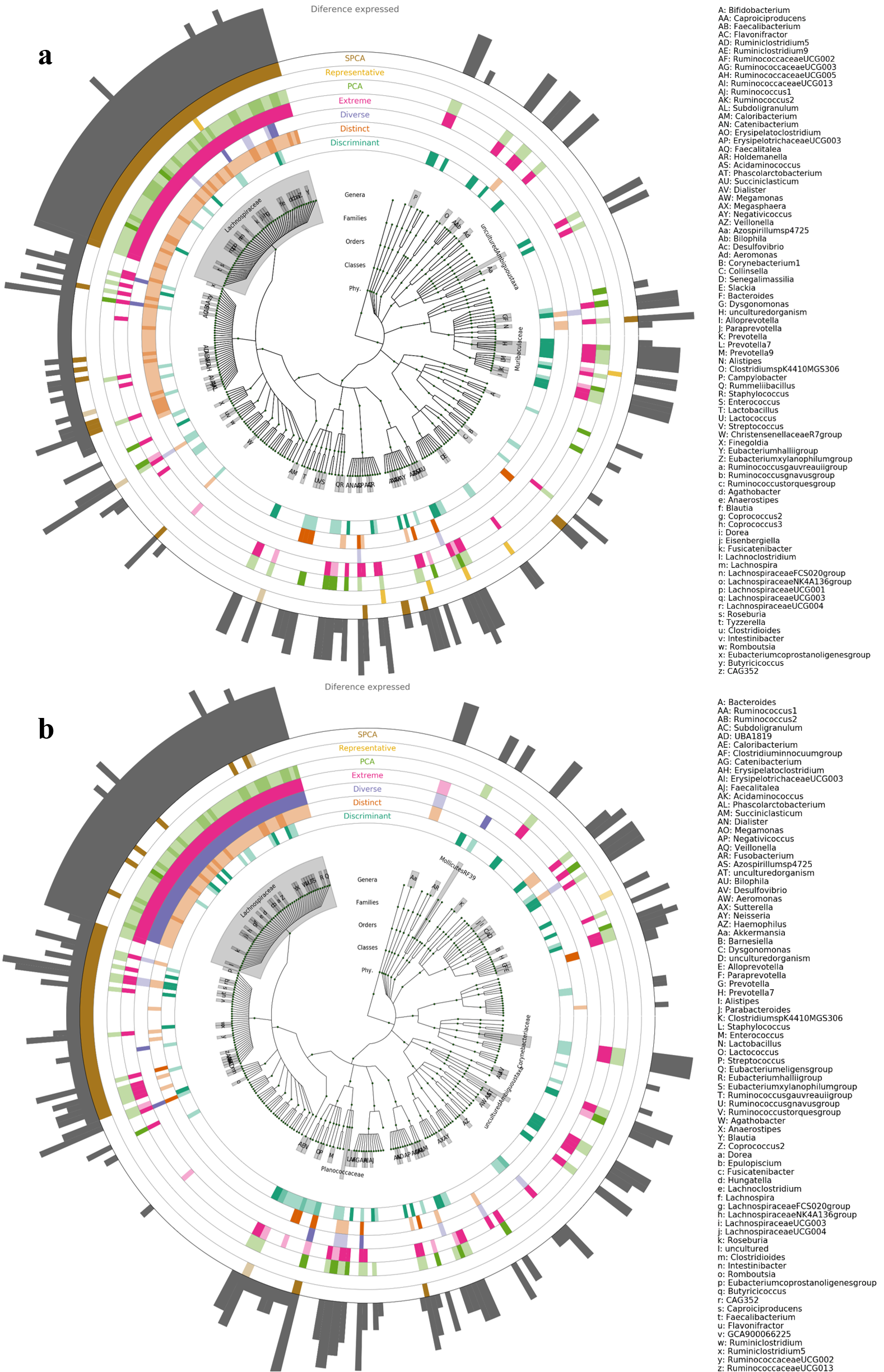

Supplement: S8 Fig — Cladograms of 16S rRNA data showing the clades more or less represented in the subsamples compared to those not selected. a) subsamples selected from DADA2 processed 16S rRNA compared to unselected samples, b) subsamples selected from Deblur processed 16S rRNA compared to unselected samples, both at subsamples of n = 20. (TIF) [file pone.0315720.s008.tif]

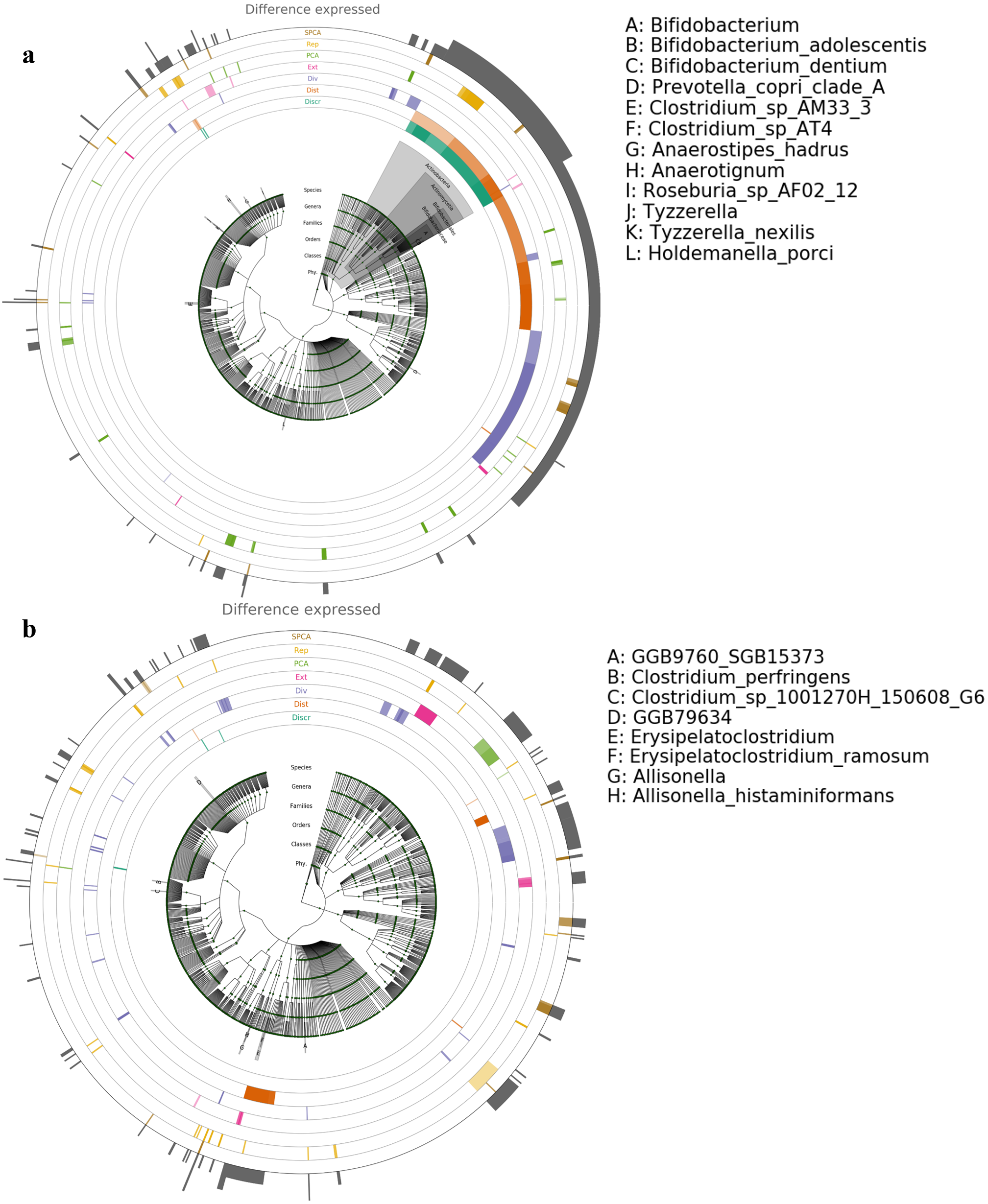

Supplement: S9 Fig — Cladograms of SM-seq data comparing presence of allergy. a) subsamples selected from ASV processed 16S rRNA, b) subsamples selected from OTU processed 16S rRNA, both at subsamples of n = 20. (TIF) [file pone.0315720.s009.tif]

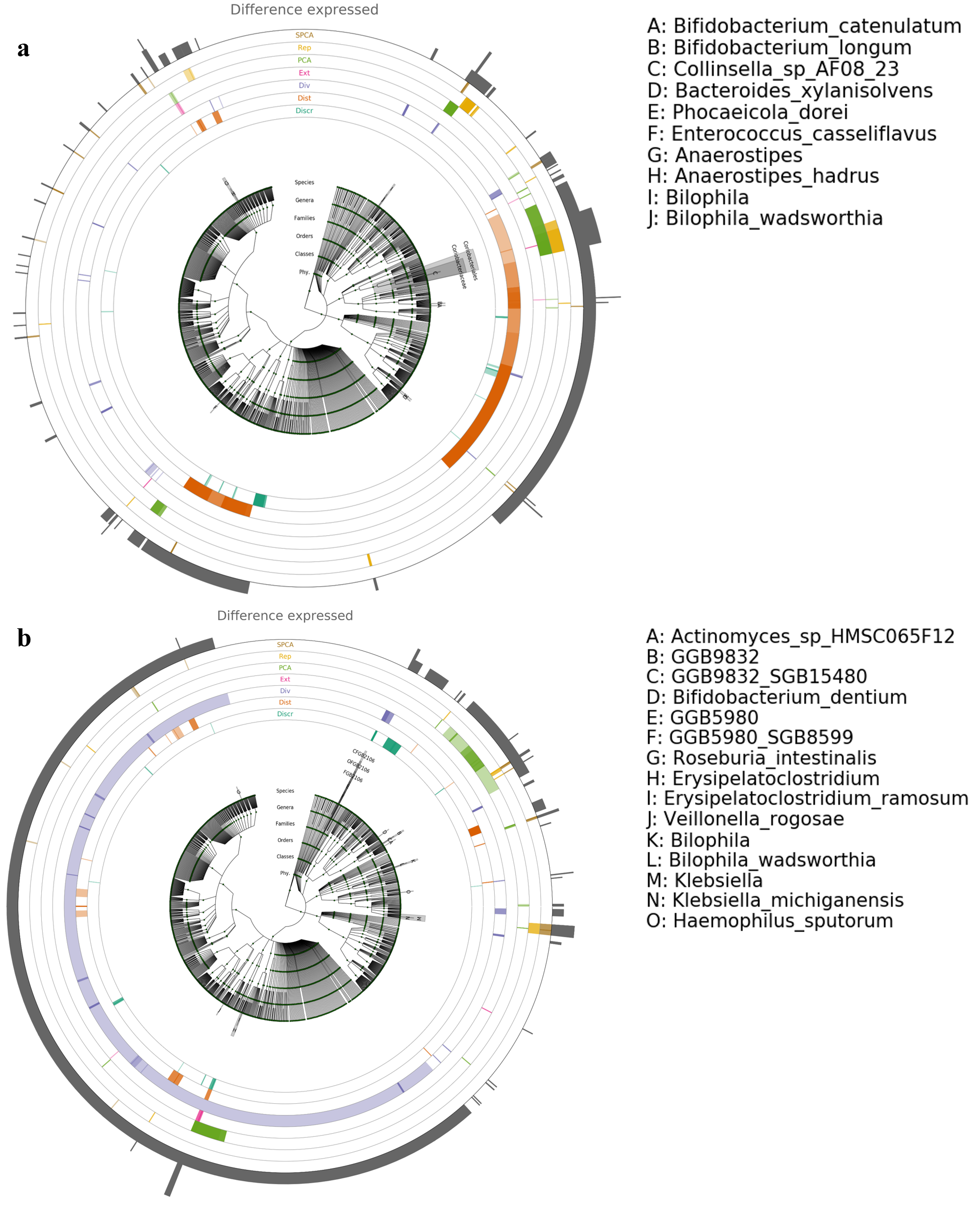

Supplement: S10 Fig — Cladograms of SM-seq data comparing presence of allergy. a) subsamples selected from ASV processed 16S rRNA, b) subsamples selected from OTU processed 16S rRNA, both at subsamples of n = 50. (TIF) [file pone.0315720.s010.tif]

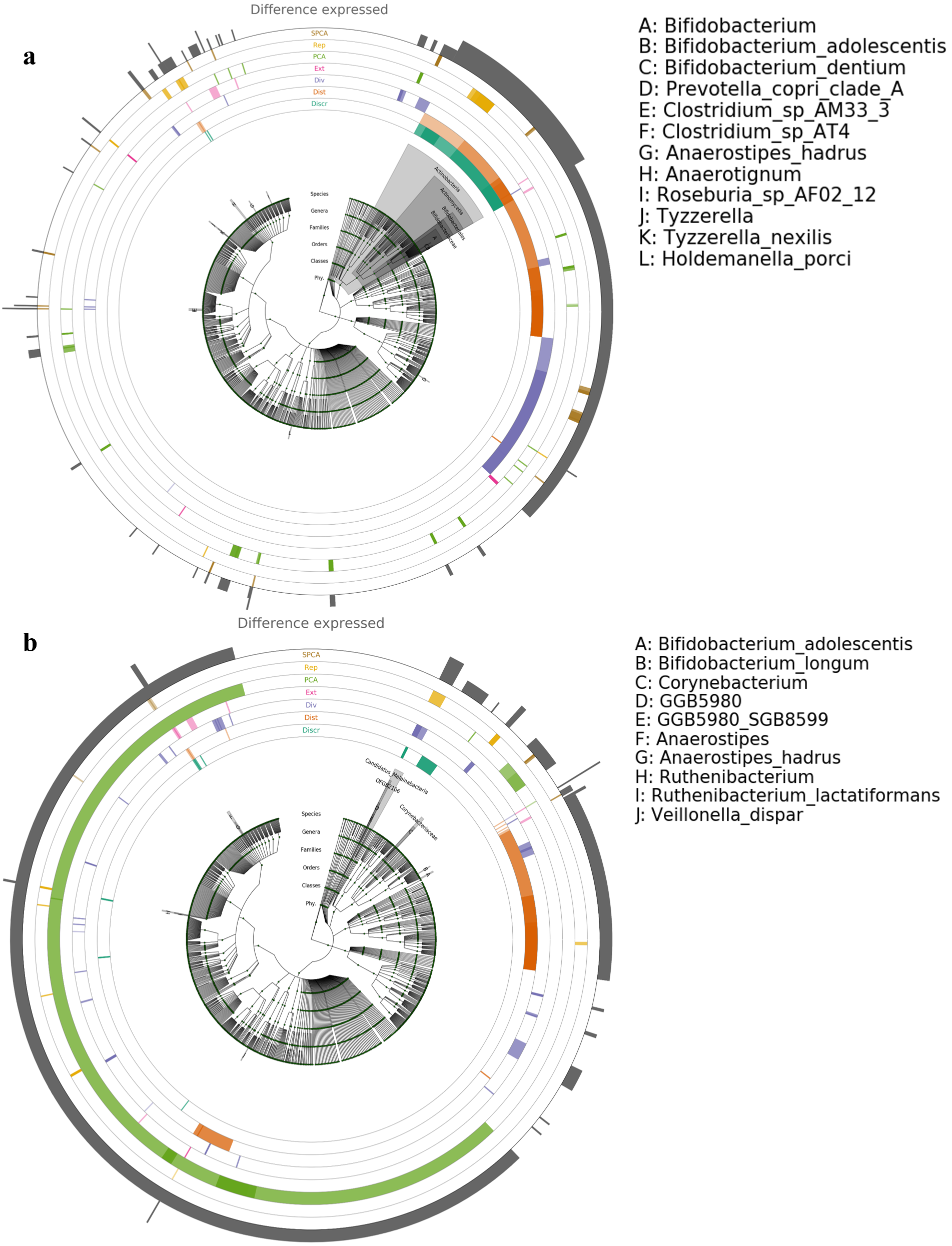

Supplement: S11 Fig — Cladograms of SM-seq data comparing presence of allergy. a) subsamples selected from DADA2 processed 16S rRNA, b) subsamples selected from Deblur processed 16S rRNA, both at subsamples of n = 20. (TIF) [file pone.0315720.s011.tif]

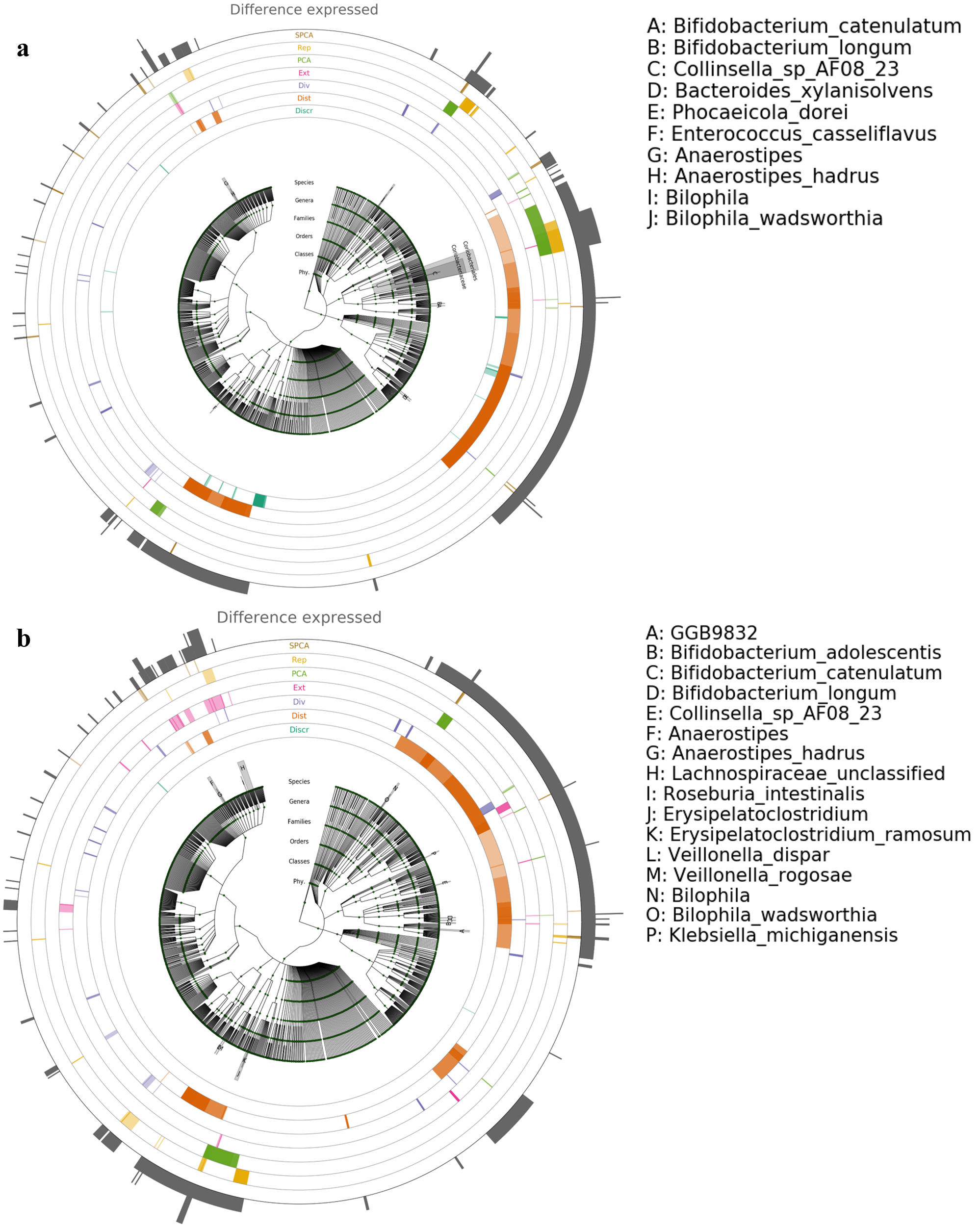

Supplement: S12 Fig — Cladograms of SM-seq data comparing presence of allergy. a) subsamples selected from DADA2 processed 16S rRNA, b) subsamples selected from Deblur processed 16S rRNA, both at subsamples of n = 50. (TIF) [file pone.0315720.s012.tif]

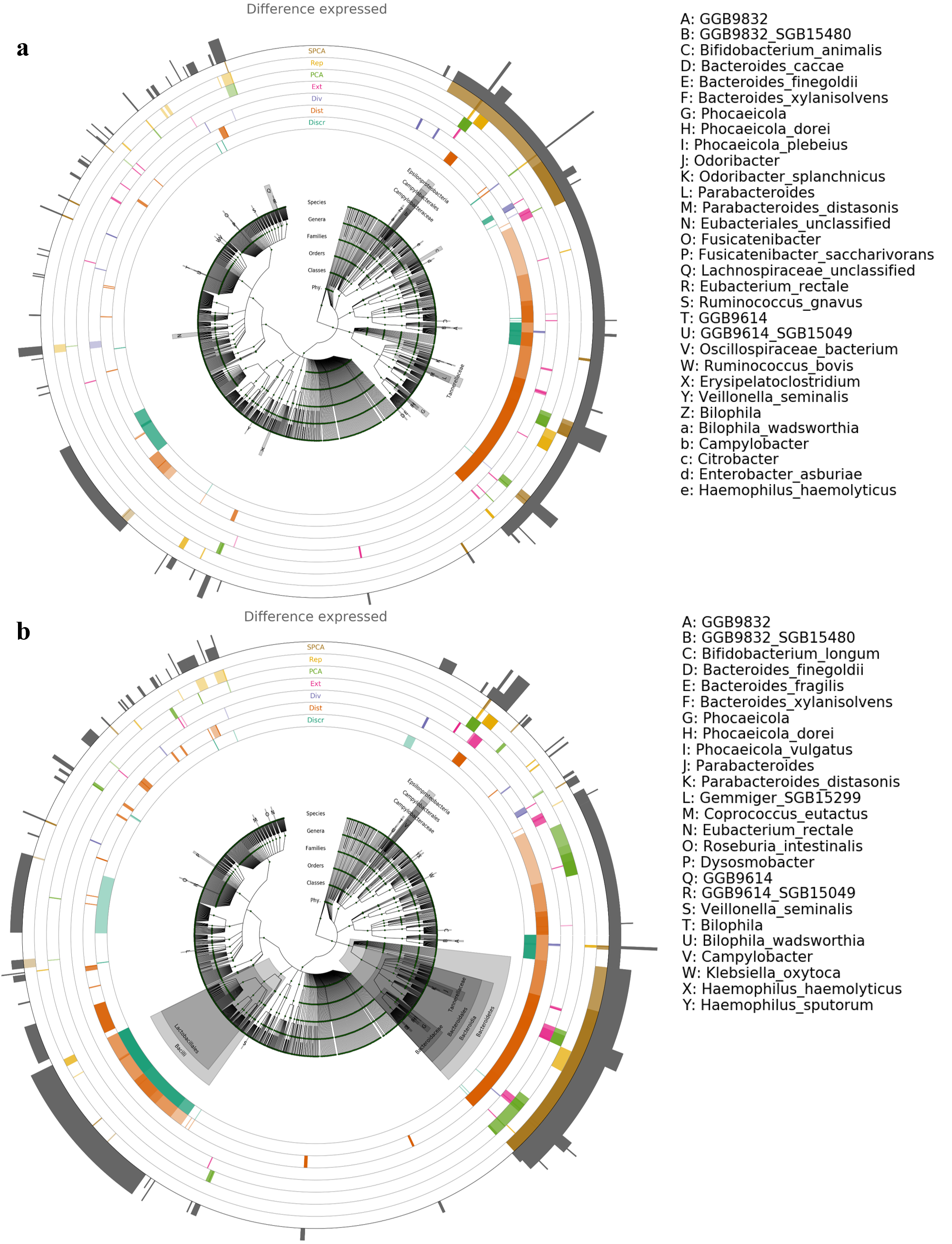

Supplement: S13 Fig — Cladograms of SM-seq data comparing presence of allergy. a) subsamples selected from DADA2 processed 16S rRNA, b) subsamples selected from Deblur processed 16S rRNA, both at subsamples of n = 100. (TIF) [file pone.0315720.s013.tif]
